# *In vivo* imaging reveals increased eosinophil uptake in the lungs of obese asthmatic patients

**DOI:** 10.1016/j.jaci.2018.07.011

**Published:** 2018-11

**Authors:** Neda Farahi, Chrystalla Loutsios, Nicola Tregay, Adam K.A. Wright, Rachid Berair, Laurence S.C. Lok, Daniel Gillett, Ian Cullum, Rosalind P. Simmonds, Charlotte Summers, Anna Wong, Chandra K. Solanki, John Buscombe, Pee Hwee Pang, Arthikkaa Thavakumar, A. Michael Peters, Christopher E. Brightling, Alison M. Condliffe, Edwin R. Chilvers

**Affiliations:** a Division of Respiratory Medicine, Department of Medicine, University of Cambridge School of Clinical Medicine, Cambridge, United Kingdom; b Institute for Lung Health, NIHR Respiratory Biomedical Research Unit, Leicester, United Kingdom; c Department of Nuclear Medicine, Addenbrooke's Hospital, CUHNHSFT, Cambridge, United Kingdom; d Division of Anaesthesia, Department of Medicine, University of Cambridge School of Clinical Medicine, Cambridge, United Kingdom; e Division of Clinical and Laboratory Investigation, Brighton and Sussex Medical School, Brighton, United Kingdom

To The Editor:

Eosinophils play an important pathogenic role in pulmonary and systemic conditions, including eosinophilic asthma and eosinophilic granulomatosis with polyangiitis.^1^, ^2^ Although progress has been made in understanding the mechanisms responsible for the activation of these cells, existing biomarkers of eosinophilic inflammation are indirect and/or invasive and do not always correlate with tissue eosinophilia. Hence, there is a need to develop noninvasive biomarkers of tissue eosinophilia. We have previously demonstrated the capacity of single photon emission computed tomography (SPECT) to quantify neutrophil uptake into the lungs of patients with chronic obstructive pulmonary disease.^3^ We sought to determine whether this methodology could be used to quantify eosinophil kinetics and pulmonary uptake, which may differ among diseases characterized by eosinophilic inflammation. In particular, the role of the eosinophil in asthma with obesity, a distinct asthma endotype associated with increased severity,^4^ is controversial. We hypothesized that injection of radiolabeled eosinophils, coupled with SPECT/CT, would reveal changes in eosinophil kinetics in patients compared with healthy volunteers.

To determine the initial distribution of eosinophils, and to ensure that the reinjected cells had not been activated, planar gamma camera imaging was performed following injection of technetium-99m–labeled eosinophils. These scans were performed in healthy volunteers, patients with asthma, and patients with focal eosinophilic inflammation (Fig 1, *A*; see Tables E1 and E2 in this article's Online Repository at www.jacionline.org). The initial transit of radiolabeled eosinophils through the lung was similar in all subjects (Fig 1, *B* and *C*). We generated a “first-pass” transit curve to calculate the time taken for the initial bolus of eosinophils to transit from the right ventricle across the pulmonary circulation (see Fig E1 in this article's Online Repository at www.jacionline.org). This value was constant across all study groups (Fig E1, *B*). The 45-minute blood recovery values (Fig 1, *D*) did not differ between the study groups and, importantly, were comparable to published levels for unmanipulated radiolabeled eosinophils from healthy volunteers, indicating that the reinjected eosinophils were nonactivated.^5^ As predicted,^5^, ^6^ circulating eosinophils accumulated rapidly within the liver and spleen, most likely within known marginated intravascular pools (Fig 1, *E*-*G*).

**Fig 1:**
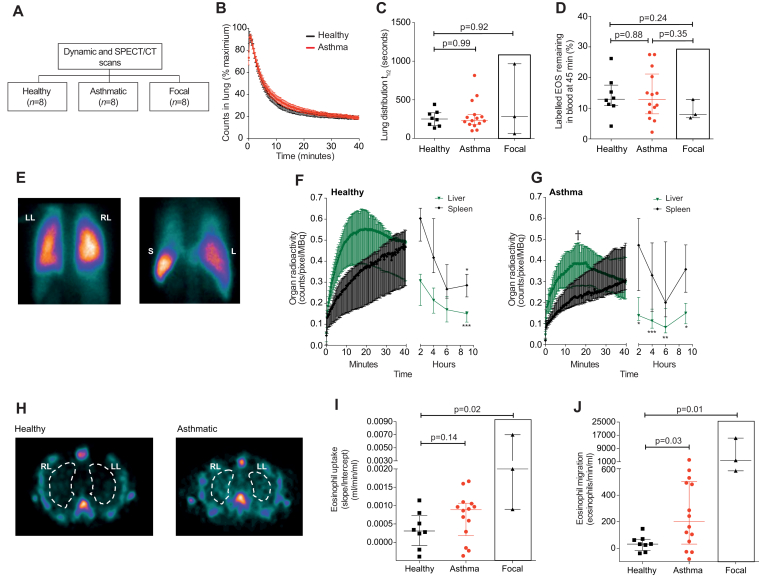
Early organ distribution and lung uptake of technetium-99m–labeled eosinophils. **A,** Participants in the scanning protocols. **B,** Time-course of radioactivity in the right lung following reinjection of technetium-99m–labeled eosinophils. **C,** Half-life of technetium-99m–labeled eosinophil activity in the lungs. Median with interquartile range (Mann-Whitney *U* test). **D,** Proportion of technetium-99m–labeled eosinophils remaining in the blood 45 minutes after reinjection. Median with interquartile range (Mann-Whitney *U* test). **E,** Gamma camera image 5 minutes *(left)* and 40 minutes *(right)* after reinjection of technetium-99m–labeled eosinophils in a healthy volunteer. Posterior images show accumulation in the right lung *(RL)*, left lung *(LL)*, liver *(L)*, and spleen *(S)*. **F** and **G,** Distribution of radioactivity in the liver (green) and spleen (black) following reinjection of technetium-99m–labeled eosinophils. Data show median with interquartile range in healthy volunteers (Fig 1, *F*) and patients with asthma (Fig 1, *G*); *P < .05, **P < .01, and ***P < .001 compared with peak liver or peak spleen radioactivity (Kruskal-Wallis with Dunn posttest). †*P* = .009 compared with healthy liver radioactivity at 17 minutes (Mann-Whitney *U* test). **H,** Transaxial SPECT images. Images show accumulation in the RL and LL (outlined by white lines) 6 hours after reinjection. Pulmonary uptake **(I)** and pulmonary migration **(J)** of technetium-99m–labeled eosinophils. Median with interquartile range (Mann-Whitney *U* test).

To measure the time-dependent uptake of radiolabeled eosinophils from the blood into the lungs (“pulmonary uptake”), serial images were acquired using SPECT/CT (Fig 1, *H*) and Patlak-Rutland analysis undertaken (see Fig E2 in this article's Online Repository at www.jacionline.org). As shown in Fig 1, *I*, pulmonary uptake was significantly increased in patients with focal eosinophilic inflammation (0.002 mL/min/mL; *P* = .02) compared with healthy volunteers (0.0003 mL/min/mL). There was a trend toward increased pulmonary uptake in patients with asthma (0.0008 mL/min/mL; *P* = .14). However, conversion of this rate of *uptake* to an absolute whole lung eosinophil *migration* value by multiplying with the peripheral blood eosinophil count revealed the full extent of eosinophil accumulation by the lungs; patients with focal eosinophilic lung inflammation and subjects with asthma had higher rates of eosinophil migration compared with healthy volunteers (32 eosinophils/min/mL) (Fig 1, *J*). The extent of eosinophil migration in patients with asthma did not correlate with lung function or fractional nitric oxide (Feno) (see Figs E3 and E4 in this article's Online Repository at www.jacionline.org).

To determine whether this technique could reveal differences in eosinophil kinetics between asthma endotypes, we stratified patients with asthma as obese (body mass index ≥30 kg/m^2^) or nonobese (body mass index < 30 kg/m^2^) (Fig 2, *A*; see Table E3 in this article's Online Repository at www.jacionline.org), and compared their eosinophil uptake. As shown in Fig 2, *B*, pulmonary uptake was increased in obese patients with asthma (0.001 mL/min/mL) compared with nonobese patients with asthma (0.0003 mL/min/mL; *P* = .02). This effect was not explained by an increase in the early retention of eosinophils in the lung because the first-pass mean transit time was faster in eosinophils of obese patients with asthma compared with eosinophils of nonobese patients with asthma (*P* = .038) (Fig 2, *C*). Furthermore, the 45-minute postinjection eosinophil recovery values and peripheral blood eosinophil counts were also similar (see Fig E4, *A* and *B*, in this article's Online Repository at www.jacionline.org). In a parallel but separate group of subjects with asthma (Fig 2, *D*), stratified in an identical manner to the SPECT/CT cohort, the bronchial submucosal eosinophil count was significantly elevated in obese patients with asthma compared with nonobese patients with asthma (*P* = .024) (Fig 2, *E* and *F*). The epithelial eosinophil count, sputum eosinophil count, peripheral blood eosinophil count, Feno levels, and IgE levels were not significantly different between patients with asthma with and without obesity (Figs E3 and E4).

**Fig 2:**
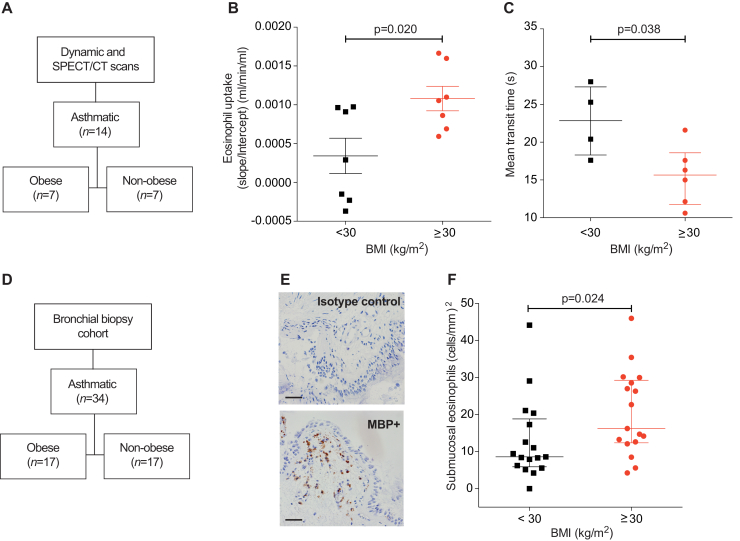
SPECT/CT quantification of pulmonary eosinophil uptake and bronchial biopsy eosinophil counts in obese and nonobese patients with asthma. **A,** Participants in the scanning cohort. **B,** Pulmonary eosinophil uptake of patients with asthma, stratified by body mass index as obese or nonobese. Mean ± SEM (unpaired Student *t* test). **C,** Transit time across the pulmonary circulation. Median with interquartile range of 10 subjects (Mann-Whitney *U* test). **D,** Participants in the bronchial biopsy cohort. **E,** Bronchial biopsy photomicrograph from an obese patient with asthma showing IgG_1_ control *(top)* and major basic protein *(bottom)*-stained eosinophils; scale bar indicates 50 μm. **F,** Quantification of bronchial submucosal eosinophil count from patients with asthma, stratified by body mass index. Median with interquartile range (Mann-Whitney *U* test). *BMI*, Body mass index.

The impact of antieosinophil therapies on eosinophil trafficking is poorly understood. Inhaled corticosteroids are the mainstay treatment for asthma and reduce the number of eosinophils in sputum and bronchial biopsies. Biological therapies such as mepolizumab^2^ reduce blood and lung eosinophil counts, but their effects on eosinophil trafficking are unknown. We anticipate that *in vivo* imaging of eosinophils will inform the development of these therapies, and shed light on the mechanisms controlling eosinophil migration.

We quantified eosinophil uptake from the blood to the lungs and demonstrated increased eosinophil migration to the lungs in our cohort with asthma relative to healthy controls. Although eosinophil migration in part reflects the peripheral blood eosinophil count, our methodology localizes inflammation and quantifies the migration to the “whole lung” parenchyma, providing information that cannot be obtained from the blood cell count alone.^7^ We observed that eosinophil uptake is enhanced in obese patients with asthma compared with nonobese patients with asthma, in agreement with our histology study. These results challenge the current dogma that asthma with obesity is associated with noneosinophilic inflammation (demonstrated by a low sputum eosinophil count),^4^ and we propose that the bronchial eosinophil count and sputum eosinophil count are uncoupled.^8^

Our study has some limitations. First, 50% of our cohort with asthma (studied in the imaging protocol) was recruited from primary care, and hence had not undergone in-depth phenotyping, and only 3 patients with asthma had a clearly eosinophilic phenotype based on Feno and peripheral eosinophil count. Second, although we have previously published a rigorous assessment of the reinjected eosinophils,^5^ we cannot absolutely exclude subtle changes in cell function due to cell isolation and labeling.

In conclusion, SPECT/CT imaging using radiolabeled eosinophils provides evidence that eosinophil uptake can be quantified in the lungs and is enhanced in obese patients with asthma compared with nonobese patients with asthma. This finding has important implications for the role of eosinophils in obese patients with asthma and for selecting patients for targeted therapy in the context of type 2 inflammation.

## Figures and Tables

**Fig E1:**
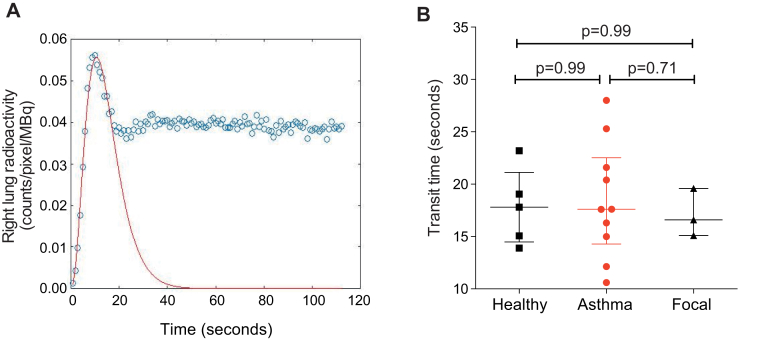
Technetium-99m–labeled eosinophil transit across the lung. **A,** Representative gamma variate function fitted to a time-activity curve. A gamma variate was fitted using a least squares residual method to simulate the first-pass time curve for eosinophils across the lung. Data from a single representative experiment (healthy control). **B,** Transit time for eosinophils to cross from the right ventricle into the pulmonary circulation for healthy volunteers, patients with asthma, and patients with focal eosinophilic inflammation. Data represent the median with interquartile range for 10 patients with asthma, 5 healthy volunteers, and 3 patients with focal pulmonary eosinophilic inflammation. *P* values calculated using Mann-Whitney *U* test.

**Fig E2:**
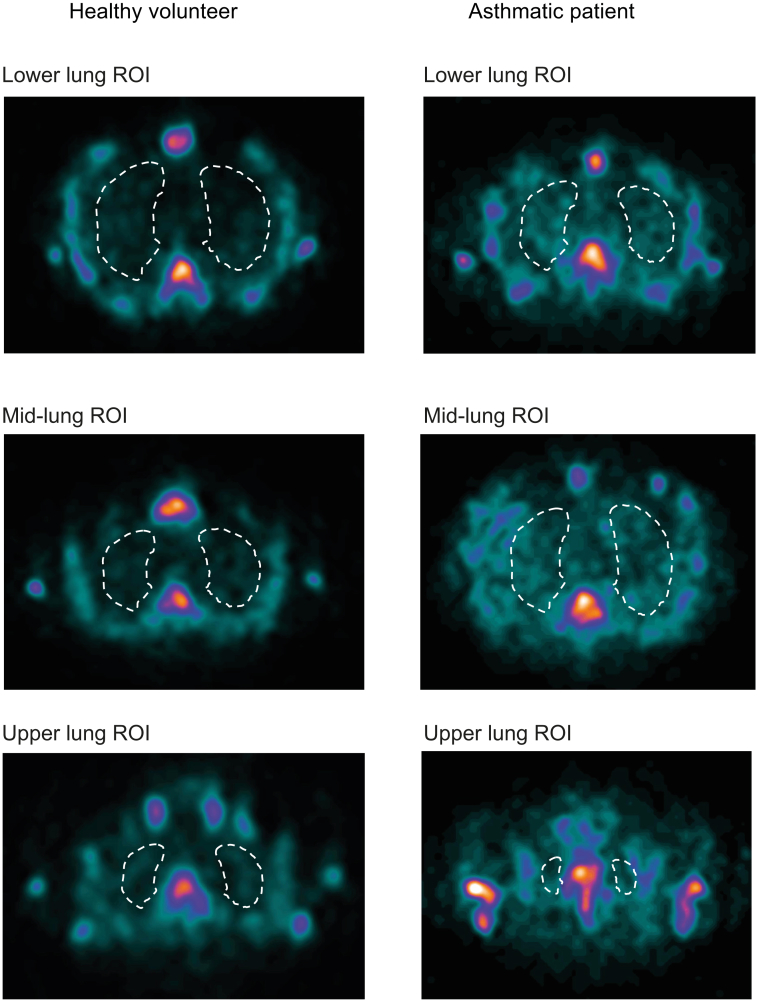
SPECT image analysis of the lung ROIs. Examples of SPECT images corresponding to lower, middle, and upper lung sections 6 hours after reinjection of technetium-99m–labeled eosinophils in a healthy volunteer *(left panel)* and a patient with asthma *(right panel)*. White lines represent lung ROI. At each time point, ROIs were drawn from sections of lung images. Each lung section was 4.42 mm thick and, depending on the size of the lung, the total number of sections drawn ranged from 10 to 15 at the early time points (45 min after reinjection) to 15 to 30 at time points 6 hours and later. The first lung ROI was drawn from the base of the lung, which was always at least 4 sections above the liver/spleen to avoid any scatter from these organs. Sequential ROIs were then drawn up to the top of the lung. When drawing the ROI, care was taken to avoid the signal from the mediastinum and bone marrow (vertebra, sternum, and ribs). For consistency, the saturation of the scan was adjusted at each time point and for each volunteer such that the vertebral body color had changed from blue to yellow, and then to the first hint of white. This saturation was chosen because it gave the optimal contrast between the lung and thoracic bony skeleton. The counts from each lung section were summed and expressed as counts/voxel before Patlak-Rutland analysis.

**Fig E3:**
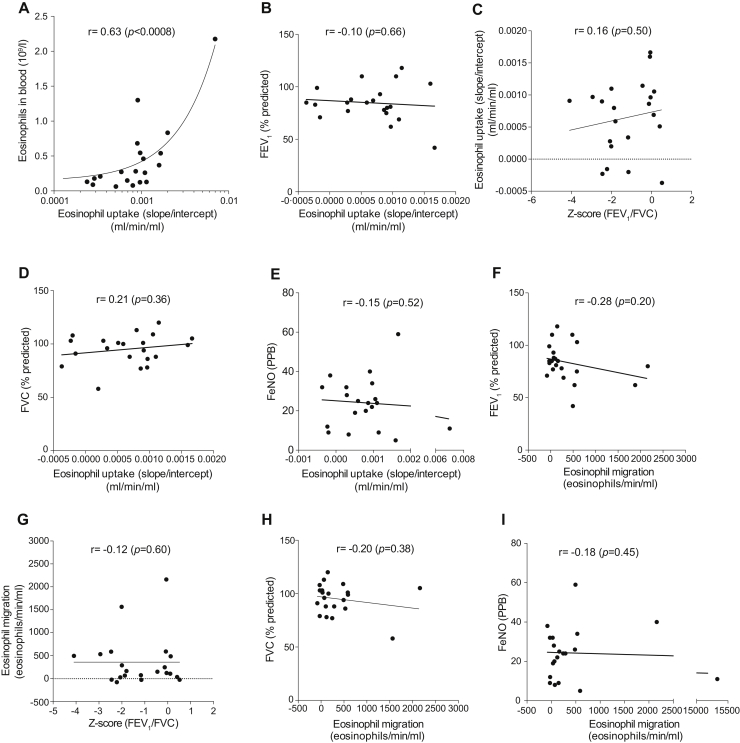
Correlations between radiolabeled eosinophil uptake or eosinophil migration into the lung with FEV_1_ (% predicted), FVC (% predicted), Feno, *Z* score (FEV_1_/FVC), and peripheral blood eosinophil count. Correlations between radiolabeled eosinophil uptake and **(A)** peripheral blood eosinophil count, **(B)** FEV_1_, **(C)***Z* score (FEV_1_/FVC), **(D)** FVC (% predicted), and **(E)** Feno. Correlations between radiolabeled eosinophil eosinophil migration and **(F)** FEV_1_ (% predicted), **(G)** FEV_1_/FVC, **(H)** FVC (% predicted), and **(I)** Feno. *FVC*, forced vital capacity; *PPB*, parts per billion. Both Feno and spirometry results were available only for 22 of 26 subjects. *P* values calculated using Spearman correlation analysis (Fig E3, *A* and *G*) or Pearson correlation analysis (Fig E3, *B-F* and *H-I*).

**Fig E4:**
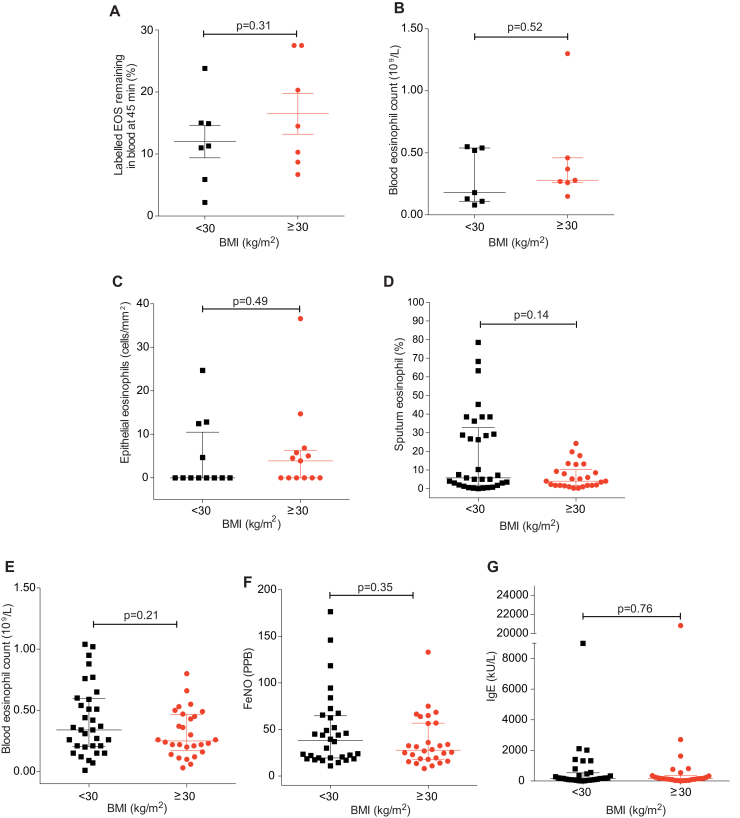
Comparison of 45-minute blood recovery values and peripheral blood eosinophil count in the obese and nonobese asthmatic SPECT/CT cohort, and peripheral blood eosinophil count, epithelial and sputum eosinophil numbers, peripheral eosinophil blood count, Feno levels, and IgE levels in the obese and nonobese asthmatic bronchial biopsy cohort. Subjects with asthma are classified by their body mass index (kg/m^2^) into nonobese (<30 kg/m^2^) and obese (≥30 kg/m^2^). **A,** Proportion of injected technetium-99m–labeled eosinophils remaining in the blood at 45 minutes after reinjection. Data represent the mean ± SEM of 7 experiments for nonobese patients with asthma and 7 experiments for obese patients with asthma. *P* values calculated using unpaired Student *t* test. **B,** Peripheral blood eosinophil counts in SPECT/CT subjects with asthma stratified by BMI as described above. Data represent the median with interquartile range of 7 subjects for nonobese patients with asthma and 7 subjects for obese patients with asthma. *P* values calculated using Mann-Whitney *U* test. **(C)** Epithelial eosinophil counts, **(D)** sputum eosinophil counts, **(E)** peripheral blood eosinophil counts, **(F)** Feno, and **(G)** IgE levels in the obese and nonobese bronchial biopsy cohort. Data represent the median with interquartile range of 12 to 33 subjects for nonobese patients with asthma and 13 to 28 subjects for obese patients with asthma. *P* values calculated using Mann-Whitney *U* test. *BMI*, Body mass index.

**Table E1:** Demographic and lung function of study subjects in the planar and SPECT/CT protocol

Characteristic	Healthy	Asthma	Focal eosinophilic inflammation
N	8	15	3
Sex: F/M	5/3	7/8	0/3
Age (y)	64 (4)	60 (2)	57 (6)
Peripheral blood eosinophil count (× 10^9^/L)	0.11 (0.08 to 0.14)	0.27 (0.16 to 0.54, *P* = .0008)	0.85 (0.72 to 2.60, *P* = .012)
BMI (kg/m^2^)∗	25 (1.46)	28 (0.82) (*P* = .04)	22 (1.76)
Feno (PPB)	14.0 (8.8 to 23.0)	25.5 (13.5 to 35.0)	11.0 (8.0 to 35.0)
FEV_1_ (% pred)	99.0 (88.0 to 112.0)	82.0 (70.5 to 90.3, *P* = .015)	62.0 (52.0 to 75.0, *P* = .017)
FEV_1_/FVC	72.0 (62.0 to 79.0)	64.5 (57.3 to 78.3)	60.0 (56.0 to 64.0)
FEV_1_/FVC *Z* score	−1.16 (−1.92 to −0.23)	−0.13 (−4.09 to −0.03)	−2.24 (−2.47 to −2.01)
Smoking status (current/previous/never)	0/2/5	0/7 (4 obese and 3 nonobese)/8 (3 obese and 4 nonobese)	1/1/1
Atopic/nonatopic	0/5	10 atopics (5 obese and 5 nonobese)/4 nonatopic (2 obese and 2 nonobese)	1/3
Comorbidities (no. of patients)	Cardiovascular disease (3), depression (1), and 4 without any stated comorbidities	Cardiovascular disease (3), gastrointestinal disease (2), hypothyroidism (1), arthritis (1), cirrhosis (1), anxiety (1), hernia (1), type 2 diabetes (1), and 7 without any stated comorbidities	Asthma (1); small joint polyarthropathy (1)

*BMI*, Body mass index; *F*, female; *FVC*, forced vital capacity; *M*, male; *PPB*, parts per billion.

Feno and spirometry results were available for 22 of 26 subjects. Data are presented as median (interquartile range) or mean (SEM). *P* values were compared to the healthy volunteer values, using Mann-Whitney *U* test or Student *t* test. Spirometric measurements were calculated using the European Community for Coal and Steel protocol, with *Z* scores from the Global Lung Initiative site (http://gligastransfer.org.au/calcs/spiro.htm).^E8^ American Thoracic Society clinical practice guidelines state that low Feno corresponds to <25 PPB, intermediate Feno between 25 PPB and 50 PPB, and high Feno >50 PPB in adults. Atopy status was assessed by a positive skin prick test result to 1 or more allergens or a clear history of seasonal allergic rhinitis.

∗ Mean (SEM).

**Table E2:** BTS Step classification and medication of participants with asthma in the planar dynamic and SPECT/CT protocol

BTS Asthma step (no. of patients in each category)	Median (range) daily ICS dose (μg BDP equivalent)	Oral daily prednisolone dose (mg)	Median (range) long-acting β2-adrenoceptor dose/d (μg)		Therapies in addition to prn salbutamol (no. of patients)
			Formoterol	Salmeterol	
Step 1 (3)	0	0	0	0	Aminophylline (1)
Step 2 (1)	400	0	0	0	None
Step 3 (6)	800 (700-800)	0	24 (24-24)	0	Montelukast (2)
Step 4 (3)	1200 (1000-1200)	0	0	75 (50-100)	Montelukast (1) Tiotropium (1)
Step 5 (2)	800 (800-800)	7.5 (5-10)	24 (24-24)	0	Tiotropium (2) Montelukast (1)

*BDP*, Beclometasone dipropionate; *BTS*, British Thoracic Society.

**Table E3:** Clinical characteristics of the study participants from whom bronchial biopsies were collected

Characteristic	Nonobese (BMI < 30)	Obese (BMI ≥ 30)	*P* value	n
Age (y)∗	51 (3)	49 (2)	.4652	61
Sex: male, n (%)	20 (60.6)	11 (39.3)	.1261	61
BMI (kg/m^2^)∗	26 (0.36)	36 (0.84)	<.0001	61
Daily inhaled corticosteroid dose (μg/d)∗†	1267 (101)	1504 (98)	.2384	61
Submucosal eosinophils (cells/mm^2^)	8.58 (5.91-18.84)	16.23 (12.37-29.27)	.0238	34
Epithelial eosinophils (cells/mm^2^)	0 (0-10.5)	3.9 (0-6.3)	.4919	25
Sputum eosinophil count (%)	5.8 (1.9-32.8)	4 (1.8-10.2)	.1444	59
Peripheral blood eosinophil count (10^9^/L)	0.34 (0.21-0.60)	0.25 (0.17-0.47)	.2134	61
ECP (ng/mL)	31.9 (19.5-54.0)	26.8 (14.6-55.2)	.5377	56
Feno (PBB)	38.3 (19.5-64.8)	27.8 (18.1-56.9)	.3512	60
Total IgE (kU/L)	172 (77-440)	158 (90-352)	.8120	59
Atopic/nonatopic	29/4	26/2	.6781	61
BTS Asthma step	4 (4-4)	4 (4-5)	.0927	61

*BMI*, Body mass index; *ECP*, eosinophil cationic protein; *PPB*, parts per billion.

Data from 33 nonobese patients and 28 obese patients based on a BMI threshold of 30 are presented as median (interquartile range) unless otherwise stated; *P* values calculated using Mann-Whitney *U* test or, for categorical data, using a Fisher exact test.

∗ Mean (SEM).

† Beclomethasone dipropionate equivalent.

## Methods

### Planar and SPECT/CT imaging of radiolabeled eosinophils

#### Participants

The study was approved by the Cambridgeshire 2 Research Ethics Committee (09/H0308/119) and the Administration of Radioactive Substances Advisory Committee of the United Kingdom (83/3130/25000); all subjects gave written informed consent. The subjects comprised 8 healthy individuals, 15 patients with asthma, and 3 patients with focal pulmonary eosinophilic inflammation (eosinophilic pneumonia, ANCA-positive vasculitis, and IgG_4_-related disease) (Tables E1 and E2). Asthma was diagnosed by a physician on the basis of symptoms, and patients with asthma were classified according to British Thoracic Society Step classification.

#### Eosinophil labeling

Eosinophils were isolated from 160 mL of autologous venous blood as described previously.^E1^ The eosinophils were labeled with technetium-99m–hexamethylpropyleneamine oxime (GE Healthcare, Buckinghamshire, UK), before reinjection into volunteers. The ex vivo preparation time was 3.75 hours. Blood was collected at intervals up to 9 hours postinjection and radioactivity measured in a gamma counter. The injected activities of the radiolabeled cells were not significantly different between the healthy individuals and patients with asthma (data not shown).

#### Dynamic, static, and SPECT/CT imaging

Volunteers were positioned in a double-headed SPECT/CT camera (GE Discovery 670, GE Healthcare), fitted with low-energy, parallel-hole collimators. After bolus intravenous injection of technetium-99m–labeled eosinophils (mean, 120 MBq; range, 46-199 MBq injected/subject), the activities in the chest and upper abdomen were recorded as previously described.^E1^ SPECT images were acquired over 20 minutes at 45 minutes, 2, 4, 6, and 9 hours following reinjection. A CT scan was performed at the end of the 45-minute SPECT acquisition for anatomical coregistration with SPECT. To generate time-activity curves, regions of interest (ROIs) were drawn over the right lung, the liver, and spleen using Xeleris software (Version 3.1, GE Healthcare). Mean counts per pixel or voxel in these ROIs were recorded and corrected for physical decay of the radionuclide. To calculate the first-pass transit time of eosinophils, a gamma variate fit was generated from the mean counts per pixel or voxel in the lung.^E2^ Gamma variate fit was performed on 15 of 22 subjects (Fig E1), because it required a minimum number of data points between 0 and 120 seconds after reinjection of the radiolabeled cells.

#### Patlak-Rutland analysis

Peripheral blood samples were taken at 2, 4, 6, 8, 10, 15, 30, 45, 90, 120, 240, 360, and 540 minutes postinjection and radioactivity measured in a gamma counter. ROIs were drawn over the lung on transaxial sections of the SPECT images at each time point (Fig E2) and average counts per voxel for each lung determined. Patlak-Rutland analysis was used to measure pulmonary eosinophil uptake per unit pulmonary eosinophil distribution volume by dividing the plot gradient by the intercept, as previously described.E3, E4, E5 Total eosinophil migration per unit pulmonary distribution volume was calculated by multiplying the eosinophil uptake by the average peripheral blood eosinophil count of each subject. These measures of eosinophil blood/lung gradient are independent of the administered activity.

### Quantitation of submucosal eosinophils in obese and nonobese patients with asthma

#### Participants

Data were obtained from the pretreatment investigations including bronchial biopsies of 61 subjects participating in a randomised, double-blind, placebo-controlled trial of fevipiprant, a prostaglandin D2 receptor 2 antagonist.^E6^ All subjects provided written informed consent. The study was approved by the Leicestershire, Northamptonshire, and Rutland Research Ethics Committees (11/EM/0402) and the UK Medicines and Healthcare Products Regulatory Agency. Details of the patient characteristics are provided in Table E3.

#### Sputum, blood, and Feno sample collection

Induced sputum (n = 59), blood (n = 61), endobronchial biopsy (n = 34), and Feno (n = 60) were collected from visit 3 of the pretreatment phase of the fevipiprant trial.^E6^ Absolute eosinophil numbers in induced sputum and blood were determined as described.^E6^ A cross-sectional analysis of this data, comparing asthma patients with and without obesity, was performed and presented here for the first time.

#### Measurement of submucosal eosinophils

Biopsy specimens were embedded in glycol methacrylate and 2-μm sections stained with hematoxylin and eosin and antieosinophil major basic protein (Clone BMK-13, Monosan, Uden, The Netherlands) as previously detailed.^E7^ Eosinophils were expressed as cells/mm^2^ of lamina propria and as cells/mm^2^ of epithelium.

#### Statistical analysis

Blood eosinophil recovery values were calculated as described previously.^E1^ Statistical analyses were performed using GraphPad Prism (6.0d, San Diego, Calif) as detailed in the figure legends. Data were tested for normality using the Shapiro-Wilk test. For nonparametric data, a Mann-Whitney *U* test was applied and for parametric data a Student *t* test was used. For categorical data, a Fisher exact test was used. A *P* value of less than .05 was considered significant. Values are expressed as mean ± SEM or the median and interquartile range.
